# Social Immersion for Women After Repair for Obstetric Fistula: An Experience in Guinea

**DOI:** 10.3389/fgwh.2021.713350

**Published:** 2021-09-03

**Authors:** Alexandre Delamou, Moussa Douno, Patrice Bouédouno, Sita Millimono, Thierno Hamidou Barry, Vandana Tripathi, Moustapha Diallo

**Affiliations:** ^1^Centre National de Formation et de Recherche en Santé Rurale de Maferinyah, Forécariah, Guinea; ^2^Africa Centre of Excellence (CEA-PCMT), University Gamal Abdel Nasser of Conakry, Conakry, Guinea; ^3^Engenderhealth, Conakry, Guinea; ^4^Hôpital Préfectoral de Kissidougou, Kissidougou, Guinea; ^5^Engenderhealth, New York, NY, United States

**Keywords:** obstetric fistula, reintegration, social immersion, guinea, quality of life

## Abstract

**Background:** Reintegration of women after repair of their female genital fistula remains a challenge. The objective of this study was to document the medical pathway and the reintegration process of women through the “social immersion” program of EngenderHealth in Kissidougou and Labé (Guinea).

**Methods:** This was a qualitative descriptive study with 55 participants, including women seeking fistula care and stakeholders involved in the social immersion for repaired women in Kissidougou and Labé. The study included collecting demographic and clinical data of women, interviews with women before and after surgery, after social immersion, and 3 months post-discharge. Municipal officials, health providers, and members of host families were also interviewed. The study protocol was approved by the Guinea National Ethics Committee for Health Research.

**Results:** The study confirmed that obstetric fistula still occurs among women living in rural and underserved areas. Most women attended at least two to five antenatal care visits, but nine over 10 reported a tragic experience of child loss associated with the occurrence of fistula. Most of them received support from their husband/partner during referral after the obstructed labor and later in the search for treatment. Women and stakeholders reported a good experience of surgery and social immersion in both Kissidougou and Labé. About 3 months after discharge, women who were continent reported being happy with their new life compared to women discharged with repair failure and residual incontinence.

**Conclusion:** The study found the positive impacts of social immersion on the quality of life of women after fistula repair, particularly for those women who had a successful repair. The approach can be included in fistula care programs, either through direct provision or through referral to programs that can provide this service.

## Background

An obstetric fistula usually occurs when women do not have timely access to quality emergency obstetric care. Prolonged obstructed labor during delivery is the most common cause of obstetric fistula, with up to 80% of patients remaining in labor for 2 or more days (1–3). It is serious childbirth-related morbidity affecting up to 1 million women worldwide, with thousands of new cases annually, mostly in Sub-Saharan Africa (4, 5). Affected women suffer from uncontrolled leakage of urine and/or feces. In addition to genital irritations caused by the permanent leakage of urine, cervical sores and nerve damage associated with prolonged obstructed labor can lead to infertility and paralysis (6, 7).

Surgery aims to repair these fistulas and restore the biological functions of a woman, but not all repairs are successful. Despite the successful repair, stress-related incontinence occurs in 16–32% of patients because of residual damage (8). Many women, even following successful repair, bear intrauterine and/or vaginal scars with cervical damage that may be associated with pelvic inflammatory disease (6).

Obstetric fistula mostly affects women living in remote and resource-limited settings (9). The health and psychosocial consequences of this disorder and the attached stigma can deeply affect the quality of life of women with the condition (10–14). Women living with fistula are often marginalized by their families and communities and live in isolation, and they are unable to participate in the household, religious, and social activities (15). They may suffer from the societal perception that they are disgusting people or cursed women who should not be allowed to be near others (16). Divorce is also common, especially when women have residual incontinence after fistula repair (17). This leads in many cases to mental distress, expressed as depression, nervousness, disappointment, bitterness, or embarrassment (16, 17). With the quality of life severely affected, many cases of suicide attempts have been reported (18).

Women with fistula suffer long-term emotional, economic, and physical consequences. Many complain that it is traumatic to be an economic burden on their families. They suffer from an inability to talk about their experience with fistula, from shame to stigma (18, 19). This experience of isolation can be explained by the lack of social assistance, lack of information about recovery after fistula repair, and poor understanding of fistula within communities (20, 21).

Given the psychosocial and other non-medical impacts of fistula, fistula care involves not just repair services, but social support for reintegration. Rehabilitating factors such as resuming social roles as wife and mother remain one of the major concerns for women after repair (22–24).

Understanding the treatment pathway and disease experience of women with fistula seems critical to improving their living conditions, especially their social reintegration after repair. The objective of this study was to assess the importance of “social immersion” in the successful social reintegration of women receiving fistula repair in Guinea.

## Methods

### Study Setting

Guinea is a coastal West African country with an estimated population of 10.5 million people, most of whom live in rural areas (65%) and poverty (26). The country is characterized by high maternal mortality and a lifetime prevalence of obstetric fistula symptoms estimated at 0.6%, two times the reported average in Sub-Saharan Africa (25).

Guinea has developed a national strategy for the prevention and management of obstetric fistula that includes the prevention at health facility and community levels, surgical repair integrated to existing public health facilities, and reintegration of women suffering fistula (social and economic reintegration) (26).

In 2015, the Islamic Development Bank (IsDB) supported EngenderHealth, a US non-governmental organization, for the surgical repair and post-repair support of 50 women at two hospitals (Labe and Kissidougou). The women included in this study were selected from this group.

### Program Description: Clinical Services

Women who presented with fistula symptoms at EngenderHealth-supported hospitals were first assessed by trained nurses and then by a fistula surgeon to confirm fistula diagnosis and determine the eligibility for surgery. Women eligible for fistula repair then received counseling and accommodation in a social house. After surgery, women received additional counseling on surgical outcomes and postoperative care. A medical assessment was also done at the time of discharge, followed by personalized counseling about topics including when to resume activities such as sexual intercourse or physical labor, family planning, and physical rehabilitation. Based on these counseling discussions, fistula counselors at each repair hospital identified fistula clients most in need of the social immersion program before returning home.

### Program Description: Social Immersion

The social immersion program sought to help women receiving fistula services reintegrate into family life and society and progressively regain their self-esteem after receiving clinical care, through transitional support from a volunteer host family. Fistula counselors discussed the program with fistula clients to choose the place, length, and content of a host family stay. After hospital discharge, women who were selected for and accepted the social immersion program were assigned to a volunteer host family for 2–3 weeks. During this convalescence period, the women made a gradual and smooth return to normal life with the support of the host family. The role of the host family was to gradually involve the visiting woman in family activities such as cooking, eating meals together, washing, shopping, and participating in social occasions such as weddings and baptisms. During the stay, host families were honored through several special visits from Mayors or their representatives along with rural radio stations. Such visits are intended to give host families pride and motivate the community to support the initiative. Host families were interviewed about their experiences by rural radio stations, with their messages broadcast to sensitize communities on fistula.

### Study Design

This was a qualitative descriptive study.

### Study Population

The study population was women receiving fistula services and stakeholders involved in the social immersion program, including care providers, host families, and community leaders, in Labé and Kissidougou. Participants were purposively selected, and arrangement to meet them was made based on their availability. Fistula clients were especially reached in their respective communities 3 months after hospital discharge.

### Data Collection and Follow-Up

Sociodemographic and clinical characteristics of participating fistula clients, along with surgery outcomes, were extracted from medical records. In-depth interviews were conducted with women before they underwent fistula repair to describe and explore the history of their disease and their journey in search of care, assess their quality of life before the repair, and describe their perceptions and expectations of rehabilitation at the time of hospital discharge.

Women were interviewed after the social immersion stays with host families to capture their experience of the stay. In-depth interviews were again conducted with women 3 months later at the home of women to evaluate the quality of life and the context of social reintegration.

Additional interviews were conducted with providers involved in the provision of care to women at the study sites, representatives of the municipalities of Kissidougou and Labé, and members of host families involved in the social immersion program at the two partner repair sites.

### Data Analysis

Sociodemographic and clinical data of study participants were descriptively summarized as means and proportions. Qualitative interview data were de-identified and transcribed into a Word file. The transcriptions were reviewed by a different research team member for conformity. A thematic content analysis approach was used in the analysis. The most relevant emergent categories and themes were generated from the data. Textual analysis was carried out using an Excel spreadsheet.

## Ethical Considerations

Fistula clients were informed about and invited to participate in the study prior to surgery by an investigator who was not part of the medical team in charge of their clinical management. Those providing a written informed consent were included in the study. This study protocol was approved by the National Ethics Committee for Health Research of Guinea (Ref# 56/CNERS/15).

## Results

Thirty in-depth interviews were conducted with 10 fistula clients (three IDIs per woman) before fistula repair, after the social immersion stay, and 3 months after they have returned to their respective communities. An additional 45 in-depth interviews were conducted with stakeholders. In total, 75 interviews were conducted with 55 participants (Table 1).

**Table 1:** Profile of participants interviewed, Kissidougou and Labé, Guinea, 2015.

**Occupation**	**Numbers**		**Total**
	**Labé**	**Kissidougou**	
Health providers	8	8	16
Midwives	2	2	4
Matron	2	2	4
Surgeon/Physician	1	1	2
Nurses	2	2	4
Anesthetist	1	1	2
Fistula clients	5	5	10
Members of host families	12	13	25
families			
Commune leaders	2	2	4
Total			55

The mean age of the 10 fistula clients who participated in this study was 34.3 years. Their sociodemographic characteristics are presented in Table 2. At discharge, eight women had their fistula closed and dry, meaning that they were continent. The fistula of a woman was not successfully closed at discharge, and one had a closed fistula but was experiencing residual incontinence. At 3 months post-discharge, their closure and continence status had not changed. Out of the 10 women interviewed, nine reported having lost their children at the delivery leading to fistula.

**Table 2:** Selected sociodemographic and clinical characteristics of fistula clients interviewed in Kissidougou and Labé, Guinea, 2015.

**ID**	**Age**	**Marital status**	**Number of pregnancies**	**Duration of fistula (months)**	**Child status**	**Previous repair**	**Status at discharge**
K1	34	Married/ Union	6	24	Stillbirth	0	Continent
K2	35	Single	5	12	Stillbirth	1	Continent
K3	38	Married/ Union	2	61	Stillbirth	0	Incontinent
K4	30	Married/ Union	6	5	Stillbirth	0	Continent
K5	28	Married/ Union	4	20	Alive	2	Continent
L1	42	Married/ Union	9	90	Stillbirth	0	Continent
L2	35	Widow	6	12	Stillbirth	0	Continent
L3	39	Married/ Union	7	24	Stillbirth	0	Continent
L4	32	Married/Union	4	48	Stillbirth	0	Incontinent (residual)
L5	30	Divorced	2	56	Stillbirth	0	Continent

### Experience of Women With Fistula

Of the 10 women who shared their experience with fistula, five were recruited in Labé and five in Kissidougou. Most of them lived in rural areas during the pregnancy leading to the fistula and needed to travel far from their village when the labor became prolonged and complicated. A woman treated at Kissidougou repair hospital stated the following:

“*My elder brother decided to take me to the city hospital for delivery given my condition. We traveled 26 kilometers to reach the city”* (Fistula client, 38 years old, Kissidougou).

The delivery that led to the fistula occurred under difficult conditions for these women, usually after 3–5 days of suffering, resulting in stillbirth in almost all the cases (9/10).

“*The labor started in the village which is situated at the top of the mountain 20 kilometers away from the health center”* (Fistula client, 42 years old, Labé).

The duration of labor in difficult conditions led to physical weakness, while stillbirth coupled with fistula occurrence caused despair to women.

“*In Kérouané, the vaginal delivery was very difficult. I had a stillborn baby and I was completely exhausted at the end”* (Fistula client, 34 years old, Kissidougou).

“*I had a stillborn baby… I felt desperate when I realized I had urine leakage”* (Fistula client, 30 years old, Labé).

Most women were fully supported by their partners after they developed fistula. At least seven out of ten women reported that they were still living with and receiving support from their husbands after they got fistula. Some reported that their husbands were involved in the search for a cure.

“*My husband is supportive and he treats me well”* (Fistula client, 28 years old, Kissidougou).

However, some reported having been abandoned by their partner.

“*My life with my husband is sad. As soon as I got ill, he married another woman and abandoned me”* (Fistula client, 30 years old, Labé).

Most women were unable to participate in social activities such as ceremonies and prayers. These women witnessed having undergone social isolation induced by stigma, bad self-esteem, and despair, affecting their physical, mental, and social well-being.

“*I don't go to celebrations or other public places, not even to the Mosque”* (Fistula client, 42 years old, Labé).

### Providers and Women Experience of Social Immersion

Women who were eligible for and accepted the social immersion program were received by host families to help begin their social reintegration.

After the program, women had positive thoughts and dreams that included resuming family and social life, pursuing happiness as a couple, becoming pregnant, and giving birth again. For some women, the feeling was that they were very tired from all the suffering and struggle at home and after searching for care. Therefore, they wanted to rest at home and have a normal life, until they become physically appealing and feel restored.

“*Thanks to my complete healing I am now clean like everyone else. I can now pray, attend parties and go everywhere”* (Fistula client, 28 years old, Kissidougou).

“*I am happy because I can now go to church and participate in women's group meetings when my health improves”* (Fistula client, 35 years old, Labé).

“*I would be very happy if God gives me even one child. I didn't think I was going to get better, that's why I had no more hope of having children”* (Fistula client, 30 years old, Kissidougou).

Providers described the process involved in establishing these placements, such as involving local authorities and preparing host families.

“*The Mayor's team has sensitized neighbourhood leaders and families to adopt and follow women discharged from hospital”* (Surgeon, Kissidougou).

Families were contacted by the Office of the Mayor of the City encouraging them to visit women at the hospital to get to know their situation. For most families, compassion, volunteering, and solidarity led them to become a host family for women with fistula. The mayors sometimes set the example to encourage their citizens.

“*As the town mayor, I have to set an example for other families; I especially served in Faranah years ago, so I hosted a woman from Faranah”* (Mayor, Kissidougou).

No prior familial relationship existed between fistula clients and host families. Nevertheless, some families hosted women originating from their villages with the help of the office of the mayor.

Host families reported that the social immersion program constitutes a means of giving back hope and self-esteem to women with fistula by providing them with the family setting that some may have been cut off from when they had a fistula. However, the effectiveness of the program appears contingent on the success of surgery, given that incontinent women may face challenges in their stay in their host families while experiencing urine leakage. Members of the host families reported that women discharged continent had a better experience of the social immersion program. Some families even encouraged women to get involved in daily activities, starting with simple activities like housework.

“*It was a pleasant stay. She gradually integrated with my family; she participated in the preparation of food for the family”* (Host family male member, Kissidougou).

This was testified by fistula clients depending on whether they were continent or not during immersion stay. Those who were discharged continent (eight over 10) reported a very good experience of the social immersion program they underwent. They enjoyed living with host families in which they felt at home. The stay made them gain self-confidence and hope.

“*The family welcomed me as if I were one of their relatives and I was happy to play with their children”* (Fistula client, 28 years old, Kissidougou).

“*My stay was pleasant since I am cured”* (Fistula client, 30 years old, Labe).

However, women who remained incontinent (two over 10) reported having lost hope because of the repair failure.

“*After the surgery, I didn't achieve my goals because I am still ill and I still feel hopeless”* (Fistula client, 38 years old, Kissidougou).

### Benefits of the Social Immersion Program

The program had a positive impact on the life of women according to health providers:

“*This program has allowed women to learn to live with families and begin their social reintegration”* (Anesthetist, Kissidougou).

According to host families, the program provided women with happiness, health, joy, new knowledge, autonomy, relief, and peace.

“*This program has brought relief to women and how to live again with people”* (Host family male member, Labé).

“*She was very worried when I saw her in the hospital, but after this stay here, she now has the smile. This is important for her return to the village”* (Host family female member, Kissidougou).

Host families also testified that they received benefits in hosting these women. According to them, these benefits included joy, new knowledge, new relationships, and honor.

“*New relationship has been created with the woman's parents”* (Host family female member, Labé).

“*Today, when I go to the municipality for an administrative document, I do not suffer because I am recognized. The Mayor has come to visit me and my name was said on the rural radio”* (Host family male member, Kissidougou).

### Reintegration Status 3 Months After Social Immersion

Three months after discharge and after the social immersion program, women with successful repairs had resumed social activities they had before fistula, in their communities.

“*I have blended in well and I am able to do anything a woman can do”* (Fistula client, 35 years old, Labé).

Their return from the repair hospital was joyful.

“*When I returned to my family, everybody was happy and I managed to adjust back to my daily life”* (Fistula client, 34 years old, Kissidougou).

Family life and relationship with relatives were described as sources of happiness and relief by women.

“*I am back in touch with my social acquaintances, my female friends now visit me and I visit them”* (Fistula client, 35 years old, Kissidougou).

Some couples were reunited following the success of the repair.

“*When my husband found out that I was cured, he came to visit my family and asked that I go home with him”* (Fistula client, 30 years old, Labé).

In contrast, women whose repair was not successful (discharged incontinent) reported that they could not resume their social activities due to urine leakage and smell. They continued to face stigma, which could be worsened by the failure of the repair.

“*Given that I could not be cured, I cannot approach people. They say that it is a curse and that I cannot do anything about it. I keep looking for a cure but I prefer to stay isolated so I do not get humiliated when I go to other people's homes”* (Fistula client, 38 years old, Kissidougou).

Women who were discharged continent seemed to be better integrated socially, while those who were not continent faced continued stigma and isolation.

### Improving Social Reintegration for Women Post-repair

To improve the social reintegration of women after fistula repair, health providers suggested providing career training and psychosocial support for these women. They also mentioned that women should be helped to know clearly that they have no fistula, to facilitate their participation in social activities.

“*We need to encourage the woman to know that she is healed and she can approach the community and act like a normal women”* (Matron, Kissidougou).

Some providers also suggested educating women and encouraging them to give birth in a health facility if they become pregnant again. Moreover, providers believed that women who have received fistula services should be involved in educating others with fistula to bring them to treatment sites. Another suggestion was to involve men (especially husbands) in the advocacy to reduce stigmatization of women suffering from a fistula or those repaired.

Host families suggested counseling for women so that they recognize they are now able to participate in social ceremonies.

“*Encouraging women to get rid of their stress, to feel healed as normal women”* (Host family female member, Kissidougou).

The other ways mentioned included teaching women small trades, extending the stay of women in host families, and awareness-raising at the community level to accommodate women after fistula repair.

“*We ask the program to increase public and community awareness so that other families could host these women. By doing so, women will know that they are loved”* (Host family female member, Labé).

Host families also suggested support for small businesses when women are back home and greater financial involvement of the authorities in supporting fistula client women as ways to sustain the program. Finally, they advised that the program should encourage host families with support such as food supplies.

## Discussion and Lessons Learned

This study highlights the experiences of stakeholders and their perceptions of social immersion and its impact on their reintegration within communities 3 months after discharge. Moreover, it also highlights their perceptions of fistula management and social immersion program and their roles in facilitating the reintegration of women in Guinea.

Overall, stakeholders had a very good opinion of the social immersion program. It was seen as a transition and a “laboratory” that prepares women for their new life, they were “reborn” and returned to social life. These views were supported by health providers and host families who also mentioned the therapy effect of the social immersion for women. For them, this stay leads to a psychological shift in women who gain self-confidence and evidence that their life has changed in a better way. It was also reported that some women were able to learn economic activities such as soap making, something that would help them get their economic autonomy upon their return to their community.

### Women's Experience of Child Loss and Fistula

Out of the 10 women interviewed, nine reported having lost their children at the delivery leading to fistula, confirming the correlation between child loss and the occurrence of fistula (1, 5, 10, 25, 27) as the condition often occurs after prolonged labor in low-resourced countries, whereas in well-resource countries, fistula occurs following surgery (iatrogenic fistula) (1, 28). These women reported physical weakness due to the duration of their labor and deep despair for both the loss of their children and the occurrence of fistula, resulting in loss of hope. Many women reported having lost weight as a result of their suffering. Also, mental health symptoms (despair, isolation, stigma, and bad self-esteem) were commonly reported. In the context of Africa, which is characterized by a strong communal life, fistula isolates women from their communities as they cannot participate anymore in the active social life they were familiar with before they experienced fistula (10, 11).

### Factors Affecting the Effectiveness of Social Immersion Programs

#### Marital Support

Most women in this study reported that their husbands were supportive of them. Support from husbands was also reported in a study in Tanzania where about half of participants described their husbands as “supportive caregivers” (29). Although divorce or abandonment of women suffering from fistula has been widely reported in the literature (11, 23, 29–32), recent reports from Guinea (27) and Cameroon (33) showed that more than two-thirds of women were still married or in the union at the time of reception at the repair hospital. This might be the result of sensitization campaigns conducted in the country to reduce stigma about fistula, and it could then constitute a leverage point for considering the involvement of partners of women in future programs.

On the other hand, it might be expected that women are not in the same need of the social immersion program depending on their marital status at discharge and the support received from their husbands and relatives during fistula. Studies have reported that women whose partners were not supportive during fistula were unwilling to go back to their marital home (29). In such situations, the social immersion program could be prioritized for those women who cannot/do not want to go home. However, most women interviewed in this study had a positive opinion of the program irrespective of their marital status. This might be due to the small sample used in this study.

#### Repair Success

Interviews with women revealed that the quality of their experience is related to their closure and continence status after surgery and at discharge. Women who became continent after surgery and stayed so during the social immersion process were happier than those who did not. Fistula closure provides satisfaction to women, but such satisfaction is only complete when the woman also becomes continent. This is because incontinent women will continue leaking urine with its offensive odor, resulting in marginalization and isolation (20, 32). It has been reported in the literature that repair outcomes are correlated with social reintegration, with women who are continent at discharge more likely to report better health and better family and social reintegration (23, 34, 35).

### Recommendations for Improving Social Immersion and Reintegration Programs

The findings of this study are similar to others that have reported interventions benefiting the quality of life of fistula clients which were documented (36–38). However, program stakeholders provided suggestions for improving the social immersion program, including providing food to host families to help them take better care of women during their stay, training and supporting women for their economic autonomy and reintegration, and improving fistula care, especially for those with residual incontinence. In addition, health providers and host families emphasized that helping host families better prepare for the stay of women is critical for the sustainability of the program. An additional suggestion was community awareness rising to reduce the stigma around the condition.

### Better Social Reintegration 3 Months Post-discharge

At 3 months post-discharge, the closure and continence status of the 10 women interviewed in this study had not changed. All women (eight out of 10) who were discharged continent reported significant changes in their lives. Return to family and social life prior to fistula was achieved for these women. One woman who was divorced because of the fistula was reunited with her husband. These women reported happiness, joy, and satisfaction. All of them were now able to fully participate in ceremonies, prayers, and daily activities. However, they were still cautious and had fear of returning to their “fistula life”. Only one of them had resumed sexual intercourse with her husband, while the remaining stating that they were still convalescent.

The woman with a fistula that was not closed and the one with residual incontinence were not satisfied with their current life. Their main concern was how to get their condition treated. They had no sexual life 3 months after discharge. However, the woman with residual incontinence reported more improvements in her family and social life than the one with repair failure.

## Limitations of the Study

The findings of this study represent the living experience of both repaired women and stakeholders involved in social immersion in Guinea. Although their viewpoints could be considered a reliable source of valuable lessons to learn from this program, information bias may be generated by this kind of study, given that stakeholders (especially host family members) might overemphasize the benefits of the program for women with the purpose to receive funds and other kinds of support.

## Conclusions

This study found the positive impacts of social immersion on the quality of life of women after fistula repair, particularly for those women who had a successful repair. Social immersion can be considered an entry point for the wider social reintegration necessary for the continuous well-being of women within communities after living with and being treated for fistula. In Guinea, the success of the social immersion program suggests that the involvement of community members in fistula management at all levels can be the center of a holistic approach to the care provided to this vulnerable population. Integrating lessons learned from this program and suggestions made by stakeholders involved would significantly improve fistula care in Sub-Saharan African countries where the condition remains a public health concern.

## Data Availability Statement

The raw data supporting the conclusions of this article will be made available by the authors, without undue reservation.

## Ethics Statement

The studies involving human participants were reviewed and approved by Guinea national ethics committee for health research (CNERS). The patients/participants provided their written informed consent to participate in this study.

## Author Contributions

AD, VT, and MD conceived the study. AD developed the study protocol with inputs from SM, VT, and MD. MD, PB, TB, and AD collected the data. AD developed the manuscript with MD and PB. VT, MD, SM, and TB critically reviewed the manuscript and validated the final version. All authors contributed to the article and approved the submitted version.

## Conflict of Interest

The authors declare that the research was conducted in the absence of any commercial or financial relationships that could be construed as a potential conflict of interest.

## Publisher's Note

All claims expressed in this article are solely those of the authors and do not necessarily represent those of their affiliated organizations, or those of the publisher, the editors and the reviewers. Any product that may be evaluated in this article, or claim that may be made by its manufacturer, is not guaranteed or endorsed by the publisher.
